# Initiation of Liver Transplantation in Bangladesh: Report on the First Two Successful Cases

**Published:** 2014-12

**Authors:** Mohammad Ali, Subash Gupta, S.M.A. Zafar, Mamunur Rashid, Muhd. Mustaque Husain, Hashim Rabbi, A.H.M. Tanvir Ahmed, K.M. Akhtar, Hasina Alam

**Affiliations:** ^1^Department of Hepato-Biliary-Pancreatic Surgery, Bangladesh Institute of Research and Rehabilitation in Diabetes, Endocrine and Metabolic Disorders (BIRDEM), Dhaka 1000, Bangladesh; ^2^Centre for Liver and Biliary Sciences, Indraprastha Apollo Hospitals, New Delhi, India; ^3^Department of Surgery, BIRDEM, Dhaka 1000, Bangladesh

**Keywords:** First liver transplantation, Liver transplantation, Liver transplantation initiation, Bangladesh

## Abstract

Liver transplantation (LT) is the treatment of choice for patients with end-stage liver disease (ESLD). Chronic liver disease due to many causes is prevalent in a significant percentage of the Bangladeshi population. Until recently, liver transplantation facilities were not available, and ESLD patients were dying without treatment. Liver transplantation is a complex procedure that requires integrated and organized approach by a multidisciplinary team. The initiation of liver transplantation in Bangladesh has faced many difficulties. These difficulties have been encountered and overcome in phases. We have successfully performed the first two living-donor liver transplantations (LDLTs) in Bangladesh. The recipient of the first LDLT was a 42-year man with cryptogenic cirrhosis, and the second one was a male of 35 years, suffering from HBV cirrhosis. Both the recipients and donors are doing well and relishing the prospect of a normal life. These two successful liver transplantations are milestones in the development of liver transplantation services in Bangladesh.

## INTRODUCTION

A large section of the population in Bangladesh is affected by various types of liver diseases. Many of them lead to end-stage liver disease (ESLD). Liver transplantation (LT) is the definitive treatment strategy for this condition. Until recently, LT facilities were not available in the country. Bangladesh was lagging behind neighbouring countries, and ESLD patients were left with no option but to wait for the untimely demise. Only very few were able to afford a transplantation which is relatively costly and intricate, especially the post-transplantation follow-up and management at overseas facilities. The initiation of LT was a dire necessity and considered a national issue. Recipients and donors were prepared. The transplantations were performed with detailed explanation of the procedure to the recipients and donors, risk and benefit were explained, and informed consent was taken from them. They also gave the consent of publication of photograph(s) for academic purpose. Ethical committee of BADAS (Diabetic Association of Bangladesh) approved the liver transplantation at BIRDEM Hospital, Dhaka.

### Problems of initiating LT in Bangladesh

Setting up the liver transplantation programme in a developing country, like Bangladesh, was associated with many difficulties. The most important was deficient infrastructure and extreme shortage of trained manpower. Lack of awareness of the benefits of liver transplantation among commoners or even medical professionals was another stumbling block. Team members needed to acquire technical expertise. Equipment and other facilities were grossly deficient. Superstitions, misconceptions, and fear about organ donation were also obstacles to the initiation of LT in Bangladesh.

### How the difficulties were overcome

The difficulties of initiating LT in Bangladesh were outlined, and the technical hitches were overcome in phases. The Bangladesh Institute of Research and Rehabilitation in Diabetes, Endocrine and Metabolic Disorders (BIRDEM) Hospital, Dhaka, a tertiary-care hospital, was chosen as the institution for launching liver transplantation. The Department of Hepato-Biliary-Pancreatic surgery was established at BIRDEM in 1999, with the aim of commencing liver transplantation in Bangladesh.

The department was equipped and provided with hepato-biliary pancreatic surgery services. This department acted as a pivot for training and developing manpower for the initiation of liver transplantation in Bangladesh.

A multidisciplinary liver transplantation team was formed by incorporation of concerned departments of the institute. Basic knowledge of hepatic surgery and LT was gained by dissection of the segmental anatomy of cadaveric livers. The surgical expertise of the team was augmented by vascular and biliary anastomosis of cow's liver, sheep's liver and pork-to-pork (porcine model) liver transplantation. The team received training in overseas liver transplantation centres. Academic discussions and work-ups were conducted by the medical professionals of government and non-government institutions.

Thirteen ESLD patients requiring liver transplants were evaluated. Recipients and donors were prepared by the members of the liver transplantation team, who were trained in performing LT in overseas liver transplantation centres. Team members also attended a number of the transplantation surgery procedures abroad. These procedures were closely followed up in our hospitals in Bangladesh. Moreover, living-donor kidney transplantation programme has been successfully ongoing at BIRDEM since 2004. This acted as a factor in obtaining patients’ confidence. Brochures and handouts on liver transplantation were distributed. Electronic and print media took an active part in raising awareness on liver transplantation in Bangladesh. The Organ Transplantation Act, 1999 of Bangladesh permits the deceased and living-donor transplants of liver and other organs. Mass awareness was created about the law, and people were urged to come forward and donate part of their liver as a lifesaving gesture to their relatives.

The multidisciplinary team worked hard for many years, equipped themselves, gained the necessary technical expertise, achieved the support of medical professionals and obtained the confidence of ESLD patients and the public. Two well-equipped operation theatres, a transplantation ICU, along with necessary instruments and ancillary facilities for LT, were set up at BIRDEM. The recipients and the donors were counselled, and they agreed to be the first candidates of LT in Bangladesh. To ensure success, extreme precautions were taken. The example was followed by a second transplantation, which was also a successful one.

## REPORT OF THE FIRST TWO SUCCESSFUL CASES

### Case 1: First liver transplantation

The first LDLT recipient was a 42-year old man weighing 60 kg, a known case of cryptogenic cirrhosis and refractory ascites. Model for end-stage liver disease (MELD) score was 20, with Child Turcotee-Pugh (CTP) score C.

The donor was a male of 29 years. Portal venography of the donor showed type B, trifurcation pattern anomaly of the portal vein into the right anterior, right posterior, and left main portal branches at its division.

**Figure 1.**
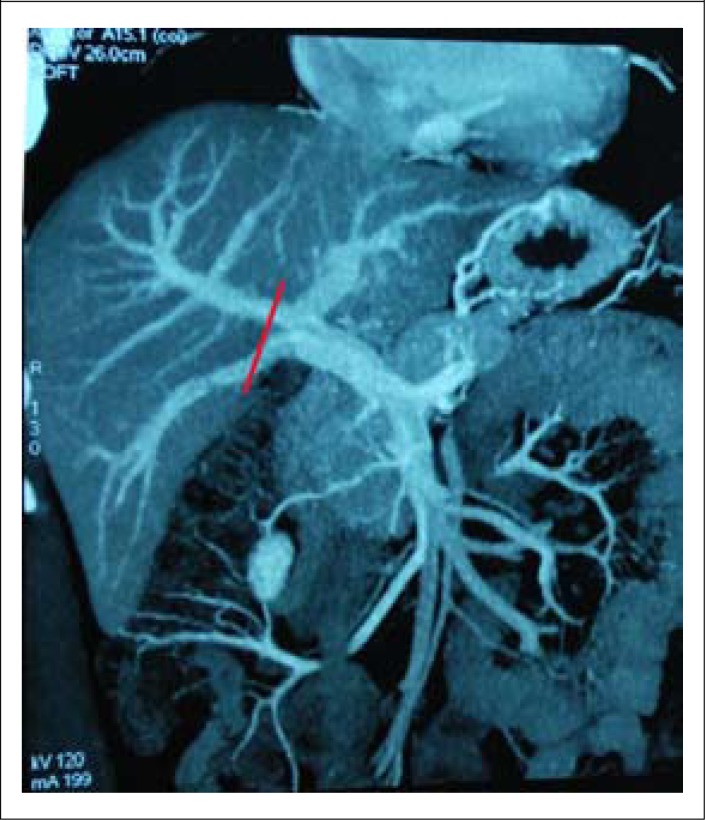
Line of portal vein division

**Figure 2.**
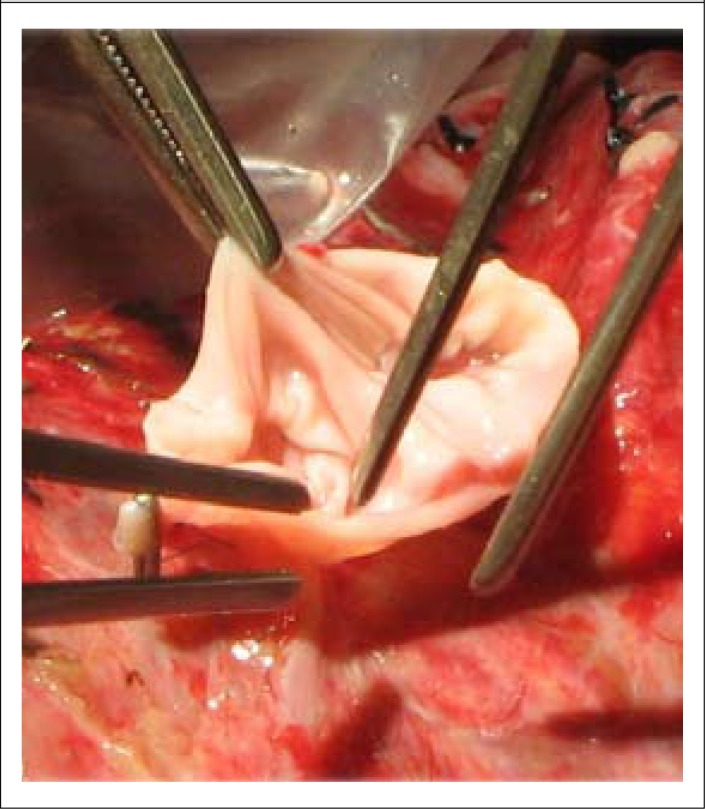
Reconstruction of portal vein

The plan was to use the right hemi-liver without MHV (Figure 1). Donor hepatectomy was performed through a right subcostal incision with midline extension. The right anterior and right posterior portal veins were divided with preservation of the left main branch. The right hepatic vein, inferior hepatic vein, right hepatic artery, and right hepatic duct were divided. The right hemi-liver without MHV was retrieved. The graft (752 g) was perfused on the back table with ice-cold HTK (Histidine-Tryptophen-α-Keto glutarate) solution. The Y graft of the main portal vein (PV) of the explant was used as an interposition graft for construction of a single lumen for anastomosis (Figure 2).

Recipient's hepatectomy was performed, and a temporary end-to-side portocaval shunt was established. Right hepatic and inferior hepatic veins were anastomosed to the inferior vena cava (IVC). The reconstructed single portal vein was anastomosed to the PV of the recipient. Graft hepatic artery with right hepatic artery (RHA) and duct-to-duct anastomosis were performed. An intra-operative doppler study demonstrated vascular patency and satisfactory perfusion of the graft.

### Case 2: Second liver transplantation

The second LDLT recipient was a 35-year old man weighing 70 kg, diabetic, with ESLD due to HBV infection. His CTP grade was C, with MELD score 15. The donor was a 38-year old male.

**Figure 3.**
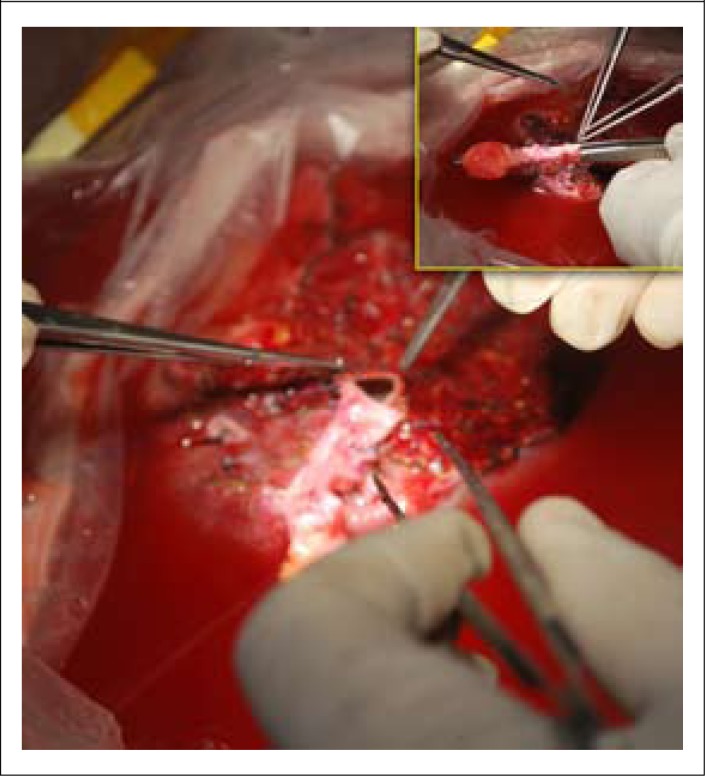
Interposition graft of MHV

The right lobe with partial MHV was retrieved (735 g) as a liver graft. An interposition graft (umbilical vein of explant) was anastomosed with the MHV on the back table preparation (Figure 3). The rightlobe liver graft was implanted orthotopically following recipient's hepatectomy. The right hepatic vein (RHV), inferior hepatic vein (IHV), and MHV extension graft were anastomosed to the IVC and the graft portal vein to recipient portal vein. End-to-end choledocho-choledochostomy and graft hepatic artery to recipient RHA were anastomosed. Intra-operative doppler study showed satisfactory vascular flow on revascularization.

## RESULTS

The donors and recipients of both transplants had an almost uneventful recovery. They have been followed up for more than three years in the case of the first transplant and more than two years in the second.

Both recipients were on a standard immunosuppressive protocol and received methyl prednisolone at the time of implantation and were maintained with oral prednisolone. The other drugs were mycophennolate mofetil (Cellcept) and tacrolimus (Prograf), tapered over six months. Entecavir monotherapy was used in the second recipient. The recipients are leading a normal life with adequate immunosuppressive agents and precautions.

## DISCUSSION

Liver transplantation has gained worldwide acceptance as an effective treatment strategy in ESLD. The programme is also rapidly advancing in the developing countries. Although liver transplantation is a demand of time and national necessity, it was associated with many difficulties in Bangladesh at the beginning. Deficient infrastructure, scarcity of equipment, shortage of trained manpower, limited resources, regressive concepts of organ donation, and lack of awareness of the benefits of liver transplantation among the public and even in medical professional circles were among the most significant obstacles (1). It was imperative to overcome all these difficulties and gain the confidence of patients and the public in order to initiate the LT programme in Bangladesh.

The Department of Hepato-Biliary-Pancreatic surgery was established at BIRDEM, with the aim of initiating liver transplantation in the country. A multidisciplinary liver transplantation team was formed; all the hurdles were identified and encountered in phases for initiating liver transplantation successfully.

Both living and deceased donor organ transplants are permitted by the Organ Transplantation Act, 1999 of Bangladesh. Organ procurement from deceased donors has not been commenced yet, which is mainly due to social and religious misconceptions.

BIRDEM is one of the prominent centres performing kidney transplants from living donors in Bangladesh. The same designed criteria were followed for liver transplants. Evaluation of a potential donor for LDLT is a difficult task. Donor's safety should be of prime importance (2,3) so that the healthy volunteer does not suffer complications.

Anomaly in the division of the portal veins may occur and is noted in 7.8% to 10.8% of the population (4). It was encountered in the first donor graft, which was technically difficult to connect the portal vein of the recipient. A graft from the portal vein of the explant was needed to reconstruct the anterior and posterior portal veins for single lumen anastomosis (5).

An estimated 15% to 40% of patients with HBV may develop ESLD (6,7). While LT is the best option, recurrence of HBV is almost universal (8,9). It has been reduced by using Lamivudine, Adefovir in combination with Hepatitis B Immunoglobulin (HBIG) (10-12). In recent years, Entecavir (ETV), a cyclopentyl guanosine nucleoside analogue has been approved as an effective monotherapy for suppressing HBV after LT (13-16).

The second recipient received ETV both before and after transplantation. His HBV DNA and HBeAg were negative, with normal liver function more than two years after the transplantation. Establishing a liver transplantation programme in a developing country, like Bangladesh, is a difficult task. However, we have achieved our goal successfully.

### Conclusions

The improvement in patient's outcome has led to the expansion of liver transplantation, not just in the developed countries but in the developing world as well. Liver transplantation is a new frontier of medical science in Bangladesh. Viral and non-viral causes of ESLD dominate the hospital admissions and mortality in Bangladesh. Every year, thousands of patients with ESLD require a liver transplantation in the country. The Department of Hepato-Biliary-Pancreatic Surgery, BIRDEM, Dhaka, has worked for a long time to organize the first two successful LTs in Bangladesh. Starting a liver transplantation programme in a developing country is associated with multiple issues. There are several critical issues that ultimately determine the success of living-donor liver transplantation. Most important is the judicious selection of the recipient and safety measures for the donor. In spite of many difficulties, the organization and performance of two successful LTs at BIRDEM have so far created a positive impact and are recognized as a landmark initiative for the continuation of liver transplantation services in Bangladesh.
